# Low-Cost, Accessible Fabrication Methods for Microfluidics Research in Low-Resource Settings

**DOI:** 10.3390/mi9090461

**Published:** 2018-09-12

**Authors:** Hoang-Tuan Nguyen, Ha Thach, Emmanuel Roy, Khon Huynh, Cecile Mong-Tu Perrault

**Affiliations:** 1 International University—Vietnam National Universities at Ho Chi Minh City, Quarter 6, Linh Trung Ward, Thu Duc District, Ho Chi Minh City 70000, Vietnam; nhtuan@hcmiu.edu.vn (H.-T.N.); hatuny97@gmail.com (H.T.); hckhon@hcmiu.edu.vn (K.H.); 2 Eden Microfluidics, 75011 Paris, France; emmanuel.roy@eden-microfluidics.com; 3 Department of Mechanical Engineering, University of Sheffield, Sheffield S1 3JD, UK

**Keywords:** microfluidics, microfabrication, photolithography, soft lithography, soft embossing

## Abstract

Microfluidics are expected to revolutionize the healthcare industry especially in developing countries since it would bring portable, easy-to-use, self-contained diagnostic devices to places with limited access to healthcare. To date, however, microfluidics has not yet been able to live up to these expectations. One non-negligible factor can be attributed to inaccessible prototyping methods for researchers in low-resource settings who are unable to afford expensive equipment and/or obtain critical reagents and, therefore, unable to engage and contribute to microfluidics research. In this paper, we present methods to create microfluidic devices that reduce initial costs from hundreds of thousands of dollars to about $6000 by using readily accessible consumables and inexpensive equipment. By including the scientific community most embedded and aware of the requirements of healthcare in developing countries, microfluidics will be able to increase its reach in the research community and be better informed to provide relevant solutions to global healthcare challenges.

## 1. Introduction

Microfluidics, through its essential role in Lab-on-a-chip (LOC) technologies, plays a key role in the development of rapid, quantitative, and sensitive diagnostic devices. The technology can diminish costs tremendously by reducing both analysis time and the amount of reagents used. Currently, the most compelling application of LOC technologies is in the early and accurate detection of infectious agents in developing countries where resources are severely limited. However, microfluidic research and development is often limited to countries with significant resources for research since current prototyping methods for microfluidic devices are costly and access to materials is limited in low-resource settings [1]. There is often a disconnect between the design of LOC devices and the realities of field application that can be overcome by widening participation in microfluidic prototyping to low-resources setting. 

Microfluidics prototyping consists of two main steps: creation of the master mold and replication into a polymeric microfluidic prototype. Master molds are generally created using photolithography, which is a complex and expensive procedure. In the photolithographic process, high-power, collimated ultraviolet (UV) light is projected through a photomask onto a photoresist (SU-8) layer to fabricate patterns [2]. The process is often performed in a clean-room environment, which requires significant capital and maintenance costs. Cheaper alternatives to photolithography include removing the need for master molds entirely such as vinyl cutters (xurography) [3,4], laser cutting [5], shrinking polymer [6,7], polyester-toner [8], paper-wax [9], and NOA adhesive [10,11], which have been explored to create master molds and microfluidic chips but lack the superior resolution of the photolithography [1]. Even though 3D printing is also a cheap, robust, and scalable method for producing master mold [12,13,14], there are still challenges that have prevented the adoption of 3D-printing by microfluidic developers, which are resolution, throughput, and resin biocompatibility [15,16]. Attempts to lower the cost of the technique have focused on the UV lighting [17] and alternatives to chrome-plated masks, but the use of a photoresist component, SU-8, has remained constant. However, SU-8 can be prohibitively expensive and complicated to source especially for researchers in low-resource countries. The lack of a high-reproducibility fabrication method that is inexpensive, robust, scalable, and sturdy to use under harsh and resource-limited environments and simple to use is a complication for low-resource countries and prevents them in joining the microfluidics research community.

We explore in this paper an alternative to SU-8 to create a master mold rapidly, cheaply, and with reasonable resolution (100 µm thickness, 100 µm feature size) using methacrylate (MA), which exists as a type of nail polish (clear UV gel from Sina^®^, product number: 1940144, Zhejiang, China). In the early 2000s, methyl(methacrylate) (MMA) was tested as a photo-resistance for microfluidic chips with pre-polymerized MMA placed in a cavity and exposed to UV [18]. However, the volume shrinkage during molding resulted in bubble formation in the PMMA channel plates. Recent development has popularized low-shrinkage meth(acrylates) (MA) first in dental clinics and as furniture sealant and, more recently, as nail polish commonly called “gels.” With the expansion of the nail cosmetic market, cheap MA gels and UV lamp sets are now readily available globally and can be exploited to bring microfluidic research in environments previously unable to cater to the high-spec needs of photolithography. 

Once the master mold is created, it is used as a template for fabrication of the polymeric device. To date, most microfluidic prototypes are fabricated through soft lithography, which was originally popularized by the Whitesides group [19]. A pre-polymer, most commonly poly(dimethylsiloxane) (PDMS), is cured on top of the master mold and, after curing, a PDMS-negative stamp of the mold is created and bonded irreversibly to glass, which creates the chips. In recent years, it has been recognized that, while PDMS has been widely adopted in academic settings and in a few commercial products (Fluidigm, Cellectricon, etc., it presents significant disadvantages (propensity for protein absorption, difficulties in scaling up for mass production, possible contamination of cyclic silicone monomer derivatives, and reversible hydrophilicity) [20,21,22,23] that has limited the translation of prototypes into marketable products [24,25]. Alternatively, a recent thermoplastic (Flexdym™, Eden Microfluidics, Paris, France) with similar desirable properties to PDMS (optical transparency from 240 to 1100 nm, flexibility, cost-effectiveness, and high patterning fidelity) was presented as an alternative for prototyping that can be easily moved into mass-production. The polymer can be soft embossed on the master mold to replicate its features, can be surface treated for permanent hydrophilicity, and bonded without plasma treatment to glass (bonding strength 1 bar) or polymers (bonding strength up to 3 bars) [26].

In this paper, we report the use of nail polish MA gels and LED-UV light to fabricate microfluidic chips. We demonstrate that the method is able to create master molds with high accuracy and high reproducibility. We also show that the resulting master molds are compatible with two common fabrication methods in microfluidics: soft lithography with PDMS and soft embossing with Flexdym. Overall, the methods used involve minimal capital costs (<$10,000) as well as minimal training and minimal running costs, which makes microfluidics research accessible to a wider community.

## 2. Materials and Methods

### 2.1. Photomask Fabrication

Photomasks were created using two different printing methods. The first method involves using a 2400 dpi laser printer (Canon LBP3300, Tokyo, Japan) onto a transparent film (Polyethylene terephthalate (PET) A4 transparent sheet), which is commonly used for printed circuit board fabrication. To improve the contrast and homogeneity of the printed pattern, a toner aided spray (Toner Aide Enhancer, Sprayway^®^, Addison, IL, USA) was used by spraying the pattern at a distance of 15 to 20 cm and dried for 5 min, according to the manufacturer’s instructions. This spray dissolves the toner, which enables merging of neighboring ink spots and filling open spaces. This results in darker patterns. The second method involves a widely used printing called offset printing from a printing provider (Saigon 3 Printing Co., Ltd., Ho Chi Minh City, Vietnam).

### 2.2. Photolithography Process

**Figure 1:**
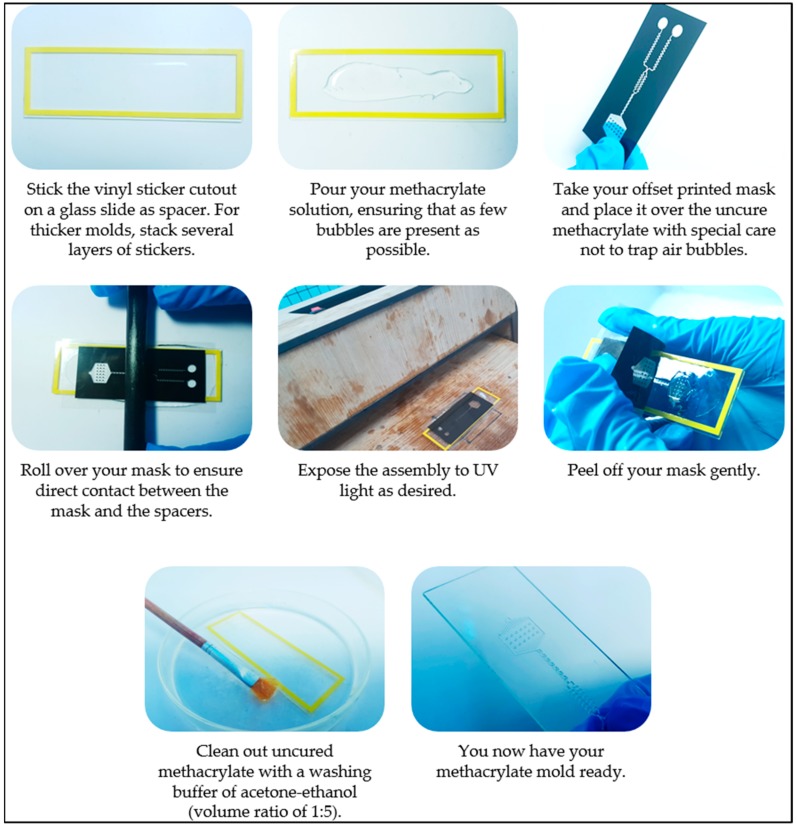
Photolithographic procedure.

The photolithographic process is described in detail below and in Figure 1.

#### 2.2.1. Methacrylate Coating

Microscopic glass slides (25 mm × 75 mm) from Ningbo Greetmed Medical Instruments Co., Ltd. (Ningbo 315040, China) were cleaned using acetone, ethanol, and distilled water. One or several vinyl stickers, 80 µm height, were stacked and attached to the glass slides to create a chamber and act as spacers. Approximately 1.2 mL of methacrylate was placed in the middle of the chamber. The photomask was then placed on top of the chamber in contact with the MA solution. An even spread was ensured by scrolling a rod along the long edge of the chamber.

#### 2.2.2. UV Exposure

The methacrylate layer covered with the photomask was exposed under 365 nm UV light from a 30-watt UV LED array of 30 LEDs ($21, UV Led Light Bead, product number: DRP-30WGH45UV365, OUMURUI, Guangdong, China). The UV LEDs were powered by a switching power supply (model: JC-480-48, JCPOWER^®^, JunChen-Tech, Guangzhou, China) that is capable of converting 220 V AC to approximately 35 V DC. 

Under UV illumination, the methacrylate crosslinked into a hardened pattern and adhered to the glass slide. The optimized UV LED-Methacrylate distance was found to be 3 cm in length and have a methacrylate thickness of 80 µm and an illumination time of 15 to 25 s.

#### 2.2.3. Washing 

After UV illumination, the photomask was peeled off and discarded. The UV exposed methacrylate layer was washed several times with a washing buffer of acetone-ethanol (volume ratio of 1:5) to remove the uncured methacrylate. For sophisticated patterns, the glass slide was dipped into the washing buffer and a paintbrush was used to gently wipe the uncured methacrylate away. Lastly, the cleaned mold was placed at 100 °C for a few minutes for drying. 

### 2.3. Molding Microfluidic Chips

To prove the compatibility of our master molds to create microfluidic prototypes, we tested them with two fabrication methods: soft lithography and soft embossing. 

#### 2.3.1. Soft Lithography

The PDMS channels were fabricated using Sylgard^®^ 184 Silicone Elastomer kit (Dow Corning, Midland, MI, USA). A 10:1 (*w*/*w*) ratio of the base and curing agent were thoroughly mixed together and poured over the methacrylate mold, which had been placed inside a Petri dish. The mixture was then degassed in a vacuum desiccator before curing the PDMS for 1 h at 90 °C. The cured PDMS was then gently peeled off the MA gels mold.

PDMS slabs were bonded with microscope slides after air plasma treatment (evacuating for 30 s, 40-s at high RF power (18 W)) by manually pressing the two parts together).

#### 2.3.2. Soft Embossing

Soft embossing was performed using the Flexdym™ and Sublym100™ (Eden Microfluidics, Paris, France) system. Flexdym™ is an alternative material to PDMS with similar mechanical and optical properties. It has the advantage of using conventional manufacturing processes to be molded (hot embossing, extrusion) [26], which permits an easy transition from prototyping to mass production of microfluidic devices. In addition, it offers better hydrophilic properties and easy bonding to most surfaces [26].

A 1-mm thick sheet of Flexdym was placed in contact with the master mold and moved into the Sublym100™. The system was set to 10 to 12 min and 105 °C. Once the molding was done, the Flexdym channels were peeled off and bonded to another Flexdym sheet by simple contact pressure. 

## 3. Results

### 3.1. Alternative to the Photomask

In developed countries, photomasks made of chrome-plated glass can be purchased from suppliers. For photomasks with feature sizes over 10 µm, the cost ranges from $100–500 depending on the masks’ size and material. There is also another option, which is the film photomask provided by companies such as Micro Lithography Services LTD with the cost only about $100 for features down to 5 µm. In Vietnam, no photomasks suppliers could be identified. We, thus, explored photomask alternatives using methods that are readily available in a resource-limited country: printing on a transparency film using an office laser printer and offset printing (at a cost of $1 per sheet with approximately 20 photomasks). 

**Figure 2:**
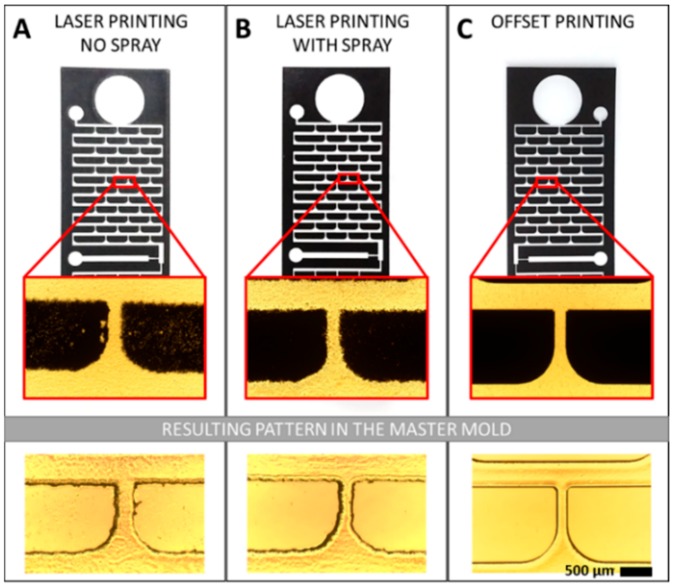
Photomasks were created using printing methods readily available for low-resource research environments. (**A**) Laser printing from an office printer presents a rough edge. (**B**) Laser printed masks sprayed a solvent-based solution are more regular but still present coarse walls and overall roughness. (**C**) Offset printing showed sharp edges and high contrasts suitable for use as a photomask.

The preciseness and resolution of the mask are critical to the quality of the lithography. As shown in Figure 2A, the mask printed using the office laser printer is not homogeneous and the edges of the pattern are coarse [27]. This is due to the printing method in which microdots of electrically-charged powder are dispensed and spaced apart to form the image on the film. The gaps let the UV light pass by leasing to an inhomogeneous curing of the methacrylate and rough channel walls. By using a toner aided spray to enhance the contrast of the photomask (Figure 2B), the small gaps can be filled, but the edges are still irregular, which leads to rough channel walls. With the offset printing technique (Figure 2C), the edges of the pattern are smooth and the printed surface is uniformly dark. The offset printing technique sequentially uses four plates with four colors (cyan, magenta, yellow, and black) and a rubber blanket to imprint the image onto the film, which results in a higher quality in the image transfer. UV curing using an offset-printed photomask resulted in channels with sharp walls and uniform cross-linked methacrylate. 

The use of an offset printed transparency represents an excellent alternative to glass photomasks in the low-resources environment.

### 3.2. Alternative to the Mask Aligner and Hg-Vapor Lamp

To overcome the lack of access to photolithography facilities, we explored the use of a LED array. In addition to their wide availability, reasonable cost, and reduced safeguards, there are several other advantages in using an LED array over an Hg-vapor lamp including the short rise time to maximum optical intensity (<300 ms) and low electrical power consumption (<6 W) [28]. The low power requirement allows the system to run on AA batteries instead of a high-voltage power supply, which is a feature that further enhances its accessibility for low-resource settings. 

**Figure 3:**
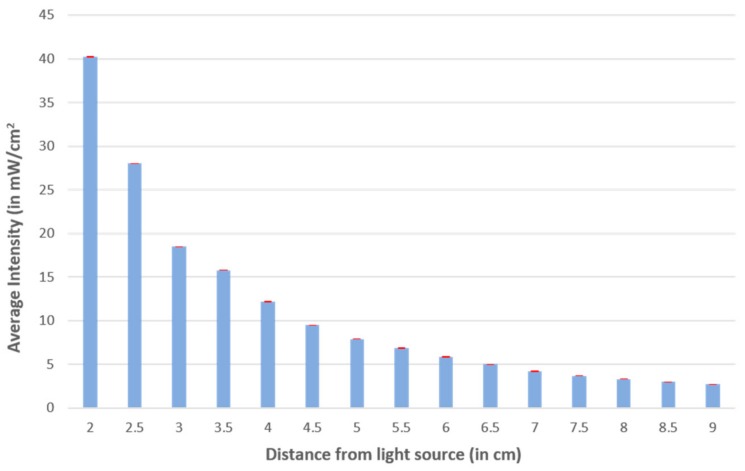
Intensity measurement in mW/cm^2^ of the exposure unit at various y distances from the source.

Since the methacrylate manufacturer did not provide specific information on the energy intensity required for cross-linking, we measured the intensity of the UV light at various distances directly under the light source (Figure 3) to link it with crosslinking performance. We found that, for a 500 µm thick layer of methacrylate, a distance of 3 cm from the UV light resulted in a good reproduction of the pattern design (Figure 3) without over-curing or under-curing, which corresponds to an energy intensity of 18.5 mW/cm^2^.

Since the array of light is not equipped to generate parallel light beams, we explored its impact on wall perpendicularity. We adjusted the distance between the light source and the chip to reduce the divergence angle and create perpendicular sidewalls. We observed a direct correlation between the perpendicular distance between the lamp and the chip and the variation of the wall angle. If the distance was too small (<1 cm), the entire layer of methacrylate was cured. We found that, for a 500 µm thick layer of methacrylate, a distance of 3 cm from the UV light for 25 s resulted in perpendicular walls (86.6° ± 1.55°) and no excess uncured methacrylate. 

**Figure 4:**
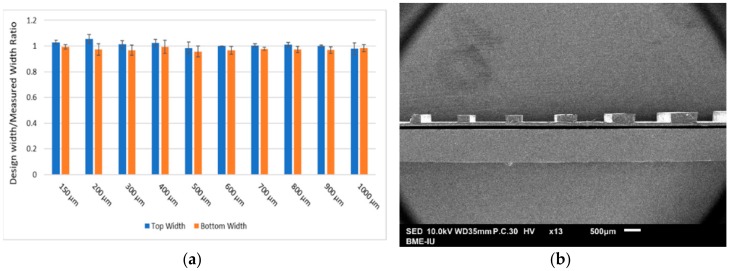
(**a**). Ratio between design width and measured width of lines of various width. All lines consisted of 500 µm thick layer of methacrylate exposed at a distance of 3 cm from the UV light for 25 s. (**b**). Cross-sectional SEM view of MA lines.

We established the reproducibility of our method by comparing lines of various width from the photomask and that of the resulting MA pattern. For a MA layer of 500 µm exposed at 3 cm from the UV light for 25 s, we found excellent agreement between design width and actual width with a correlation index R2 of 0.9992. The ratio between design width and measured width of lines ranging from 150 µm to 1000 µm was found to be within 1 ± 0.03 for all width when measured at the top of the line. When measured from the bottom of the line, the ratio was found to be closer to 0.97 ± 0.03 (Figure 4). 

### 3.3. Alternative to Spin Coating

**Figure 5:**
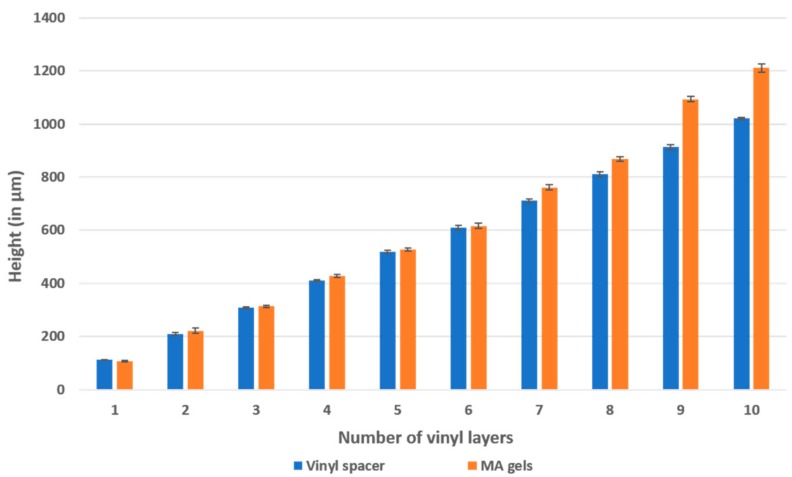
Methacrylate layer thickness according to spacer’s height.

The standard method for controlling the thickness of the photo resistive layer and achieving the micro-channels’ height commonly involves a spin coater. For low-resource setting labs that may not be able to afford a spin coater, we found that using vinyl stickers as spacers can be a good alternative method (Figure 5). By changing the height of spacers, we were able to produce microchannels in methacrylate with heights varying from 80 µm to 1 mm, which provides a wider range of thicknesses than the conventional photolithographic equivalent using SU-8. However, we noted that, as the layer of methacrylate became thicker, the microfluidics sidewall was no longer perpendicular to the bottom surface. The recorded slope angle created by the sidewall and the bottom surface at 1.3 mm thick methacrylate (exposed at 3 cm for 25 s) was found to be 115.19°. It is, thus, important to note that the thickness of the gel will influence the final geometry. 

### 3.4. Compatibility with Microfluidics Fabrication Methods

**Figure 6:**
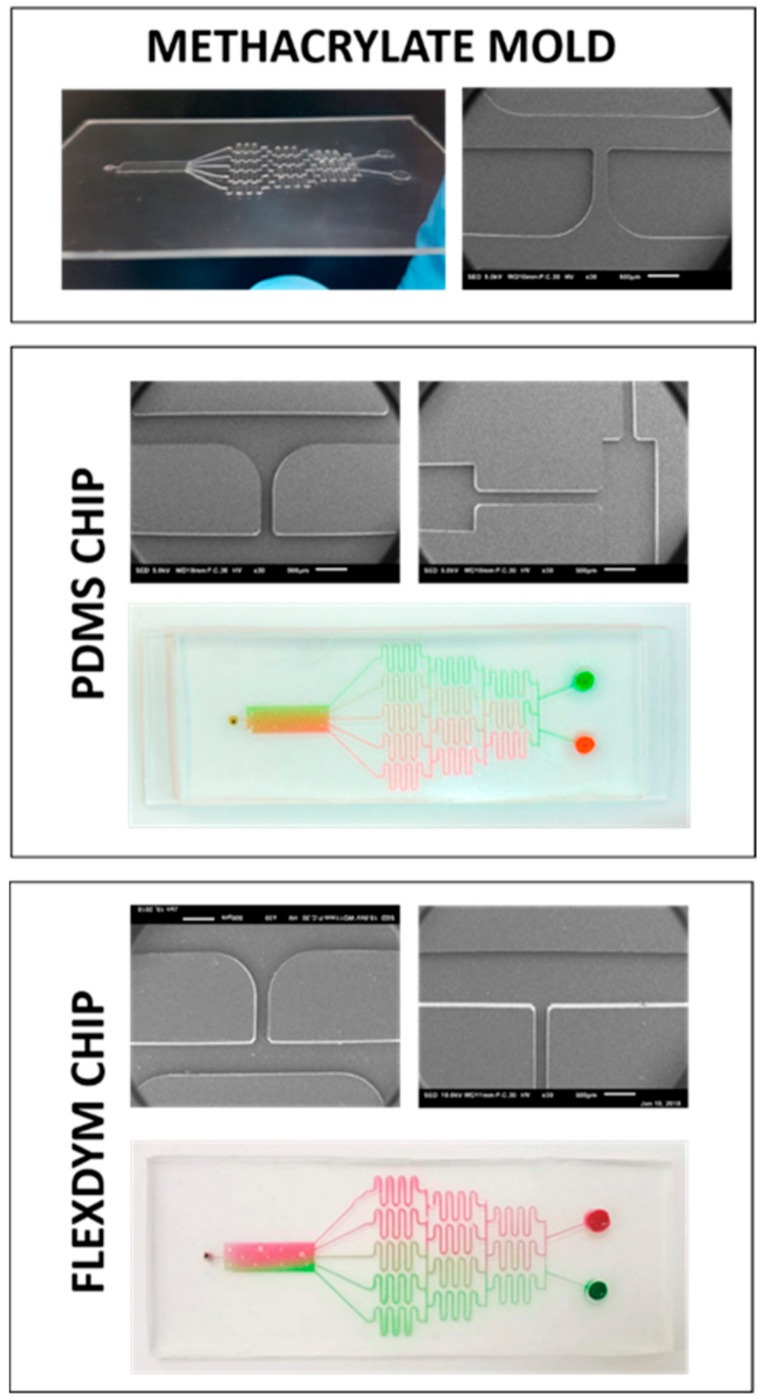
The methacrylate mold was successfully used to reproduce various shapes and design in two polymers adapted to microfluidic applications: PDMS and Flexdym.

The MA molds were used to produce the PDMS chip using soft lithography. The polymer did not adhere to the mold once cured and the patterns were reproduced accurately (Figure 6). The channels were smooth and well-defined, which validates this method for fabrication of microfluidics molds. Figure 6 illustrates several fabricated designs using the proposed method such as capillary pumps and gradient generators, which performed satisfactorily and the fluids flowed easily when tested shortly after plasma bonding.

Due to a number of its shortcomings (absorption of small molecules, manufacturing method incompatible with mass production, and reversible hydrophilicity), PDMS is often considered less than ideal for prototyping. We, thus, also tested our master mold with an alternative material, Flexdym, which uses a molding method similar to hot embossing called soft embossing. The method requires less temperature and pressure than the traditional hot embossing method (120 °C for 5 min at 5 bar). Similar to the PDMS chips, we observed high-reproducibility of the pattern and smooth walls (Figure 6). One additional advantage of the Flexdym material is that bonding does not require plasma treatment, which reduces the initial capital cost by a significant amount. Alternative methods of bonding PDMS without plasma [10,29,30] can also be used, which either adds an adhesive layer to the PDMS or uses stickers while Flexdym present the advantage of an inherently adhesive nature [26]. Lastly, the chips made with Flexdym were more hydrophilic than their PDMS counterpart and, therefore, filled more easily with the reagents.

## 4. Conclusions

In summary, we have developed an affordable, accessible process to fabricate microfluidic devices using resources available and reasonably-priced for low-resource settings.

This simple process was able to create microfluidic devices with critical feature sizes around 100 µm and channel heights up to 1 mm. Importantly, we demonstrated that high-quality patterns could be generated without specialized cleanroom equipment such as mask exposers (~$15 k) or aligners (~$50 k), spin coaters (~$7 k), and plasma machines(~$7 k), which lowers the setup cost for a microfluidic lab from ~$30,000 to $65,000 to about $6,000 (UV exposure lamp ($21) and Sublym100 system (~$6 k)). It is important to note that the consumables themselves are massively reduced from an acetate mask (~$200), SU-8 (~$400/L), and PDMS (~$200/kg) to offset printing sheets ($1/sheet), MA gel ($10/15 mL), and Flexdym (~$25/sheet of 15 cm × 15 cm). We anticipate that the ability to prototype a wide range of devices with minimal setup and running costs will accelerate the development of microfluidic devices and, most importantly, enable researchers from low-resource settings to join the community of microfluidics engineering.
